# Diagnostic yield of CT thorax angiography in patients suspected of pulmonary embolism: independent predictors and protocol adherence

**DOI:** 10.1007/s13244-014-0325-5

**Published:** 2014-04-03

**Authors:** Stefan Walen, Minke-Alie Leijstra, Steven M. Uil, Martijn F. Boomsma, Jan Willem K. van den Berg

**Affiliations:** 1 Department of Pulmonology, Isala, Dr. van Heesweg 2, 8025 AB Zwolle, The Netherlands; 2 Department of Radiology, Isala, Dr. van Heesweg 2, 8025 AB Zwolle, The Netherlands

**Keywords:** Pulmonary embolism, Computed tomography, Thoracic radiography, Diagnostic imaging, Caregivers

## Abstract

**Objectives:**

To determine the diagnostic yield of computed tomography scanning of the pulmonary arteries (CTPA) in our centre and factors associated with it. Differences between specialties as well as adherence to protocol were investigated.

**Methods:**

All patients receiving a first CTPA for pulmonary embolism (PE) in 2010 were included. Data about relevant clinical information and the requesting specialty were retrospectively obtained. Differences in diagnostic yield were tested using a chi-squared test. Independent predictors were identified with multivariate logistic regression.

**Results:**

PE on CTPA was found in 224 of the 974 patients (23 %). Between specialties, diagnostic yield varied from 19.5 to 23.9 % (*p* = 0.20). Independent predictors of diagnostic yield were: age, sex, D-dimer, cough, dyspnea, cardiac history, chronic obstructive pulmonary disease (COPD), atelectasis/consolidation, intrapulmonary mass and/or interstitial pulmonary disease on CT. Wells scores were poorly documented (*n* = 127, 13.0 %). Poor adherence to protocol was also shown by a high amount of unnecessary D-dimer values with a high Wells-score (35 of 58; 58.6 %).

**Conclusions:**

The diagnostic yield of CTPA in this study was relatively high in comparison with other studies (6.7–31 %). Better adherence to protocol might improve the diagnostic yield further. A prospective study could confirm the independent predictors found in this study.

***Teaching Points*:**

*• Pulmonary embolism is potentially life-threatening and requires quick and reliable diagnosis.*

*• Computed tomography of the pulmonary arteries (CTPA) provides this reliable diagnosis.*

*• Several independent predictors of diagnostic yield of CTPA for pulmonary embolism were identified.*

*• Diagnostic yield of CTPA did not differ between requesting specialties in our Hospital.*

*• Better protocol adherence could improve the diagnostic yield of CTPA for pulmonary embolism.*

## Introduction

Pulmonary embolism (PE) is a serious, potentially life-threatening disease that requires timely and effective treatment. Its diagnosis can be difficult, since symptoms are non-specific [1]. Current Dutch guidelines recommend a diagnostic work-up in three steps. Firstly, the clinical probability should be determined by calculating a Wells score [2]. Wells’ criteria are shown in Table 1. Secondly either the Wells-score is high (a score of more than four points) and a computed tomography scan of the pulmonary arteries (CTPA) should be made or, if the Wells score is low (four points or less), the D-dimer level should be assessed. Thirdly, a high D-dimer level (≥0.5 mg/ml) also dictates performing CTPA [2–4]. CTPA is currently regarded as the reference standard for confirming the diagnosis of pulmonary embolism. Its sensitivity is estimated between 60 % and 100 % and its specificity between 81 % and 98 % [5–8]. Studies show that the diagnostic yield of CTPA for pulmonary embolism varies between 6.7 % and 31 % [9–14]. In our teaching hospital (Isala, Zwolle, The Netherlands) approximately 1000 CTPA’s are made annually. Most of these CTPAs are requested by pulmonologists, internal medicine specialists or cardiologists. In this pilot study we tried to investigate the quality of the diagnostic work-up for pulmonary embolism in our hospital. We tried to determine whether there were differences in diagnostic yield between the requesting specialties, to find out the extent of protocol adherence (to perform the aforementioned diagnostic steps in the right order) and to assess independent predictors of diagnostic yield of CTPA. We expected a uniformly good standard of diagnostics, with a diagnostic yield conforming to figures from the recent literature, with room for improvement of protocol adherence.

**Table 1:** Wells’ criteria

Clinical criteria	Score
Clinical signs or symptoms of DVT	3.0
PE is number one diagnosis or equally likely	3.0
Heart rate greater than 100 beats per minute	1.5
Immobilization (at least 3 days) or surgery (previous 4 weeks)	1.5
Previous, objectively diagnosed PE or DVT	1.5
Haemoptysis	1.0
Malignancy (treatment current or within 6 months or palliative)	1.0
Total score	….

## Materials and methods

All patients receiving a CTPA for pulmonary embolism from 1 January 2010 to 31 December 2010 were included in this study. For all the exams a 40- or 64-slice MDCT with helical scan (Royal Philips) was used with a peak voltage of 120 kV and an exposure of 300 milliampere-second per slice. Electronic and paper medical files were searched for the CTPA outcome, clinical characteristics and relevant medical history. The Wells score was dichotomised to low (≤4) or high (>4), and D-dimer levels also to low (≤0.5 mg/ml) or high >0.5 mg/ml). The specialties were classified into five groups: pulmonologists, cardiologists, internal medicine specialists (including neurologists and anaesthesiologists/intensive care specialists), surgical specialists and mixed specialists (urology and gynaecology).

A chi-squared test was used to determine the differences in diagnostic yield between the specialties. A Student’s *t*-test for two independent groups was used to test for differences in D-dimer level between men and women. An association between D-dimer level, Wells score and the diagnostic yield was univariately tested with a chi-squared test. When appropriate, a Fisher’s exact test was used. To identify independent predictors of the diagnostic yield of CTPA, multivariate logistic regression was used. Because there was little evidence from the literature as to which factors could affect diagnostic yield of CTPA, all factors collected in this study were interpreted in this analysis. Furthermore, selecting factors based on *p* values seemed less likely to give a good model, since clues for the direction of a possible relationship were lacking [15]. Factors were differentiated in pre-diagnostic factors (patient characteristics) and complementary factors (findings on imaging techniques).

A *p* value of 0.05 or less was regarded as significant. The “Statistical Package for the Social Sciences” (SPSS), version 18.0.1 was used for statistical analysis.

## Results

**Fig. 1:**
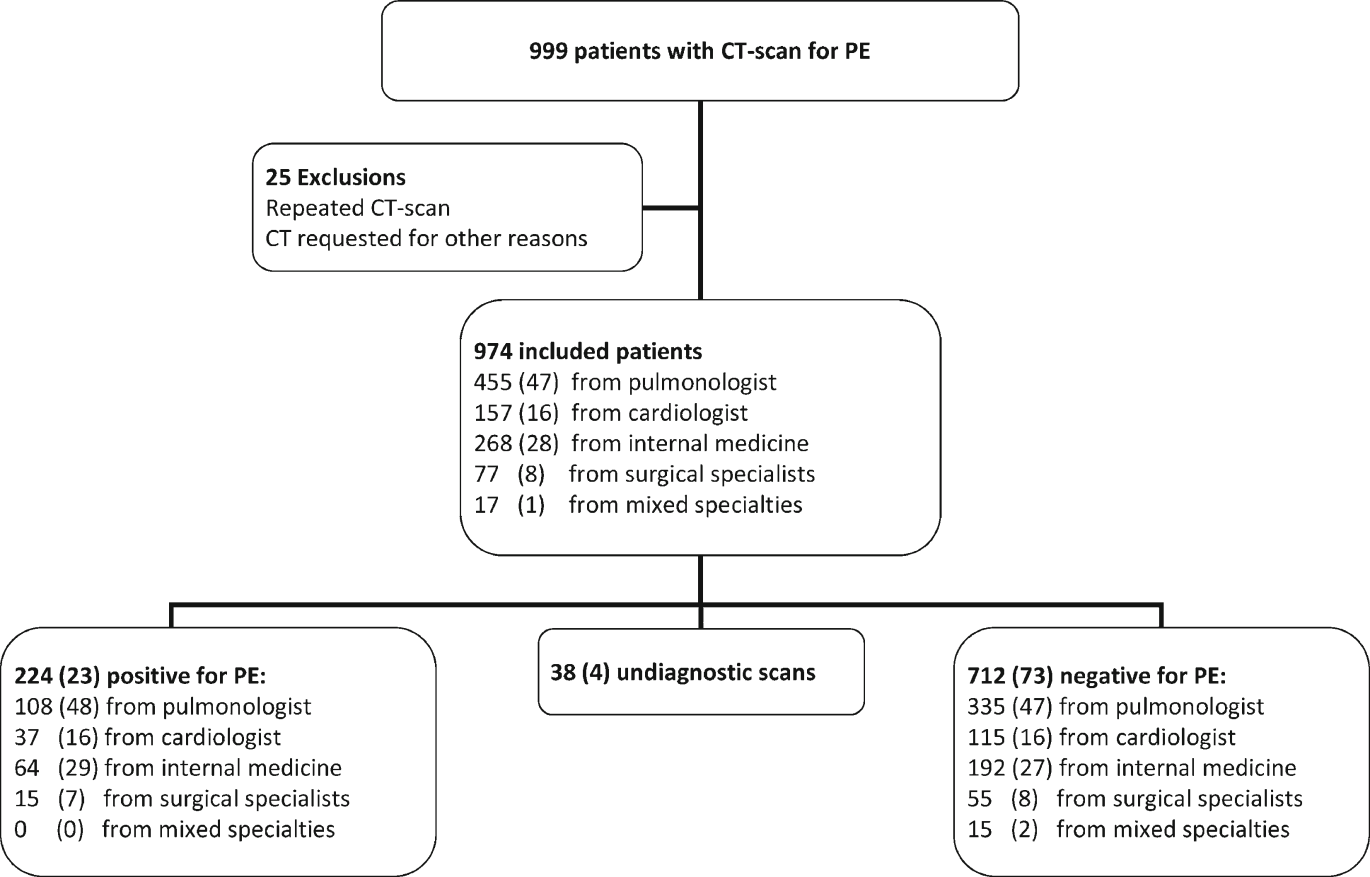
Flowchart of patient inclusion. Distribution per specialty is shown. Number (%)

Between 1 January 2010 and 31 December 2010, a total of 999 patients underwent CTPA for the suspicion of pulmonary embolism in our hospital. Of this group, 22 patients were excluded because of repeated CTPA and three were excluded because CTPA was requested for other reasons. The setting for the 974 remaining patients was as follows: 691 patients presented at the emergency department (70.9 %), 214 were inpatients (22 %) and 47 were outpatients (4.8 %). For 22 patients, the setting was unknown. Figure 1 shows the flow chart for inclusion with distribution per specialty. Table 2 shows patient characteristics. There appeared to be slightly more women than men, without a significant age difference between these groups. Men had a higher mean D-dimer level than women (3.97 vs 2.89; *p* = 0.04), and proportionally more often had a high Wells score (56 % vs 39 %; *p* = 0.06).

**Table 2:** Patient characteristics (*n* = 974)

Sex (%)	530 female (54.4)
Age distribution	≤50 years: 148
	>50 years: 382
	444 male (45.6)
	≤50 years: 83
	>50 years: 361
Average age [years (SD)]	63 (16.7)
	Female: 63 (17.8)
	Male: 64 (15.3)
Known mortality in 1 year[*n* (%)]	193 (19.8)
	Pulmonary embolism: 7
	Other cause: 58
	Unknown cause: 127
Morbidity [*n* (%)]	Malignancy 272 (27.9)
	Cardiac history 251 (25.8)
	COPD 125 (12.8)
	Surgery within past 4 weeks 132 (13.6)
Pulmonary embolism on CTPA [*n* (%)]	224 (23.0)
	Undiagnostic: 38 (3.9)

*SD* standard deviation, *CTPA* computed tomography scan of the pulmonary arteries, *COPD* chronic obstructive pulmonary disease

Pulmonary embolism was found in 224 of the 974 included patients (23 %). This group consisted of 111 women and 113 men. A scan was named undiagnostic in 38 cases (4 %), meaning the reviewing radiologist could neither confirm nor rule-out a (small, subsegmental) pulmonary embolism. The differences in diagnostic yield between the specialties were analysed without the group “mixed specialties” due to the small number of patients in this group (*n* = 17). The diagnostic yield of the other specialties varied between 19.5 % and 23.9 %. There was no significant relationship between the diagnostic yield of CTPA and the various specialties (*p* = 0.20).

Univariate analysis showed no relationship between the Wells score and the diagnostic yield of CTPA (*p* = 0.84). For the D-dimer level, however, the relationship with the diagnostic yield was significant. Analysis showed that a higher D-dimer level was associated with a higher diagnostic yield of CTPA (odds ratio 13.1; 95 % confidence interval 1.8–96.5; *p* < 0.0001).

The Wells score was documented for 127 patients (13.0 %), the D-dimer level for 601 patients (61.7 %), and both values were present for 97 patients (10 %). The Wells score was documented most frequently by pulmonologists (*n* = 100; 22 %), followed by the internal medicine specialists (*n* = 22, 8.2 %), the cardiologists (*n* = 4, 8.2 %) and mixed specialties (*n* = 1, 5.9 %). Surgeons did not document the Wells score on any occasion. D-dimer levels were documented most frequently by cardiologists (*n* = 125; 79.6 %) and pulmonologists (*n* = 352; 77 %), followed by mixed specialties (*n* = 8; 47 %), internal medicine specialist (*n* = 69; 35 %) and surgeons (*n* = 20; 26 %). In 35 out of 58 patients (58.6 %) with a high Wells score, D-dimer level was also documented. Of which 26 were requested by the pulmonologist. In case of a low Wells score, D-dimer level was missing in 5 out of 69 cases (5.2 %). An association between diagnostic yield of CTPA and various combinations of high and low Wells score and D-dimer levels could not be interpreted due to low numbers.

The low numbers of documented Wells scores made the multivariate analysis of independent predictors of diagnostic yield of CTPA that included this parameter difficult to interpret. Therefore, we chose to control the complete analyses which assessed the predictive properties of the pre-diagnostic and complementary factors for: age, sex, speciality and setting.

In Table 3, the results of the analysis of pre-diagnostic factors are shown. Chest pain, dyspnea, cough, haemoptysis, malignancy, cardiac history, Chronic obstructive pulmonary disease (COPD) and surgery in the previous 4 weeks were included in the analysis (*n* = 866) as pre-diagnostic factors. Sex was found to be an independent predictor; female sex was associated with a lower yield. A lower yield was also found for a presentation with cough, cardiac history and/or history of COPD. Higher age and a presentation with dyspnoea increased the odds of finding pulmonary embolism. There was a borderline-significant trend for chest-pain increasing diagnostic yield (*p* = 0.052).

**Table 3:** Multivariate analysis: pre-diagnostic factors affecting diagnostic yield of CTPA

Factor	OR	95 % CI	*p* value
Sex	0.641	0.460–0.894	0.009
Age	1.014	1.003–1.025	0.013
Dyspnea	1.589	1.098–2.299	0.014
Cough	0.655	0.437–0.980	0.039
Cardiac history	0.556	0.364–0.849	0.007
COPD	0.490	0.273–0.879	0.017

Significant factors are shown with an odds ratio and a 95 % confidence interval

*CTPA* computed tomography scan of the pulmonary arteries, *OR* odds ratio, *95 % CI* 95 % confidence interval, *COPD* chronic obstructive pulmonary disease

Table 4 shows the significant findings of the analysis of complementary factors. This analysis, with data of 851 patients, included the controlling factors described above and the complementary factors: alternative diagnosis on chest X-ray, findings of an infiltrate, atelectasis/consolidate, ground-glass aspect, interstitial abnormalities and an intrapulmonary mass on CT. In this analysis, sex and age were found to be similarly associated with diagnostic yield, as found in the pre-diagnostic analysis. Complementary factors that made a pulmonary embolism on CTPA less likely to find were: atelectasis/consolidate and interstitial abnormalities.

**Table 4:** Multivariate analysis: complementary factors affecting diagnostic yield of CTPA

Factor	OR	95 % CI	*p* value
Sex	0.705	0.509–0.978	0.036
Age	1.013	1.003–1.024	0.014
Atelectasis/consolidate on CTPA	0.616	0.394–0.964	0.034
Interstitial abnormalities on CTPA	0.360	0.204–0.633	<0.0001
Intrapulmonary mass on CTPA	0.480	0.247–0.931	0.030

Significant factors are shown with an odds ratio and a 95 % confidence interval

*CTPA* computed tomography scan of the pulmonary arteries (CTPA), *OR* odds ratio, *95 % CI* 95 % confidence interval

## Discussion

This study aimed to determine the diagnostic yield of CTPA and investigated differences in this yield between specialties. Furthermore, protocol adherence was examined. To our knowledge, it is the first study that also aimed to identify factors that could influence the diagnostic yield of CTPA. We think that it is very likely that the working method regarding the diagnostics of pulmonary embolism in our centre resembles the situation in many other hospitals closely. So, in our opinion, results might be generalised. The diagnostic yield of 23 % that we found is relatively high in comparison with values reported in the literature (6.7–31 %) [9–14]. Another study in The Netherlands found a diagnostic yield of 19.1 % [16]. The relatively high diagnostic yield could be the result of technological advances in scanning technique, leading to the discovery of more small, subsegmental pulmonary emboli, the clinical relevance of which is still controversial [17]. An example of this development is the introduction of dual-energy multidetector CT, which, in preliminary studies, shows promising results [18]. The differences in diagnostic yield between the specialties (19.5–23.9 %) in this study were not significant, which could be an indication of a uniformly high quality of diagnostic work-up for pulmonary embolism in our hospital, although this is not properly documented, as is shown in the following paragraph. According to protocol, for every patient with a suspicion of pulmonary embolism, a Wells score should be calculated. This study found however, that for only was 13.0 % the Wells score documented. Proportionally most Wells scores were documented by pulmonologists (22 %), followed by internal medicine specialists (8.2 %). It is likely that in daily practice this score is being calculated more often than it is documented. The D-dimer level, on the other hand, is being documented in many more cases, namely in 61.7 % of the patients in this study.

As earlier stated, only when the Wells score is low should the D-dimer level be assessed. High D-dimer levels dictate making a CTPA. However, in daily practice, the D-dimer level is often being requested together with standard laboratory investigations immediately when a patient presents with a suspicion of pulmonary embolism. This intends to save time, but may result in too many D-dimer level requests, and thus in unnecessary costs and unnecessary requests for CT scans. In case of a high Wells score, the D-dimer level is being requested in 58.6 % of patients, which is not according to protocol recommendations; and D-dimer level was missing for 5 out of 69 patients with a low Wells score. These results suggest that D-dimer level and Wells-score were requested and interpreted independently, which is in contradiction of the protocol, which recommends to consistently follow the three steps in pulmonary embolism diagnostics.

From the literature, little is known about factors associated with diagnostic yield of CTPA. To gain more knowledge about this, we conducted a multivariate analysis to identify potential pre-diagnostic and complementary factors. Unfortunately, the most important multivariate analysis in this study could not be controlled for Wells score and D-dimer level, since there were too many missing values for both of these factors together. The performed analysis showed that higher age, male sex, dyspnea, atelectasis/consolidate on CT and/or intrapulmonary mass on CT were independently associated with higher diagnostic yield of CTPA. There was a borderline significant trend for chest pain. It was less likely to find a pulmonary embolism on CT in presence of the following factors: female sex, cough, cardiac history, COPD and/or interstitial abnormalities. Univariate analysis raised the suspicion that a higher D-dimer level was also associated with a higher diagnostic yield.

The presence of an alternative diagnosis for the symptoms of the patients with cardiac history, COPD, atelectasis/consolidate on CT and/or interstitial abnormalities on CT could be the explanation for the lower yield of CTPA in these patients. The influence of age could be attributed to the fact that with increasing age the incidence of pulmonary embolism is also increased [19]. The association with higher diagnostic yield in patients with chest pain and dyspnea is an indication that these findings correctly raise a suspicion of pulmonary embolism [20, 21]. Female sex seemed to protect against finding pulmonary embolism. To explain this, two possible reasons can be given. Firstly, in our study significantly lower D-dimer levels and almost significantly lower Wells scores were found in females. Secondly, in our study, more females than men were undergoing CTPA, while the literature showed that there are reasons to believe that the incidence of pulmonary embolism is equal among the sexes [2, 22–24]. It may be the case that doctors are sooner inclined to perform CTPA in (young) female patients because of risk-factors not being scored in the Wells table, e.g. contraceptives or pregnancy, even though this is speculative.

In every retrospective study the reliability of data can be a problem, because data from letters or notes made at the time of presentation can be misinterpreted. Probably, some of the CTPAs were requested for reasons other than suspicion of pulmonary embolism and some CTPAs could have been falsely classified as positive. The largest problem was, however, posed by the fact that the Wells score was known for such a limited number of patients, which made some of the analysis, as described in the results section, difficult to interpret. This caused a problem in determining the true independent influence of Wells score and D-dimer on diagnostic yield of CTPA.

This study shows a relatively high diagnostic yield of CTPA in our hospital, without differences in requesting specialties, which could indicate uniform good quality of pulmonary embolism diagnostics in our centre, although this is not properly documented. Adherence to protocol proves to be subject for improvement, especially since the Wells score and D-dimer levels appeared to be requested and interpreted independently. Better adherence to the protocol could improve diagnostic yield and lower costs. Furthermore, reduction of unnecessarily requested CTPA leads to a reduction in radiation exposure. In order to make sure the three steps in diagnostics are followed, it might be considered to develop a requesting system in which respectively a D-dimer and a CTPA can only be requested after a Wells score is determined and documented.

A prospective study will be conducted to confirm the independent predicting factors of the diagnostic yield of CTPA found in this study. This could aid the development of new clinical assessment tools. Moreover, in this manner the influence of the Wells score and D-dimer can also be better determined.
